# Follicle‐stimulating hormone promotes renal tubulointerstitial fibrosis in aging women via the AKT/GSK‐3β/β‐catenin pathway

**DOI:** 10.1111/acel.12997

**Published:** 2019-06-26

**Authors:** Kun Zhang, Lin Kuang, Fangzhen Xia, Yi Chen, Wen Zhang, Hualing Zhai, Chiyu Wang, Ningjian Wang, Yingli Lu

**Affiliations:** ^1^ Institute and Department of Endocrinology and Metabolism, Shanghai Ninth People's Hospital Shanghai Jiao Tong University School of Medicine Shanghai China; ^2^ Institute and Department of Gynecology and Obstetrics Sir Run Run Shaw Hospital, School of Medicine, Zhejiang University Zhejiang China

**Keywords:** AKT/GSK‐3β/β‐catenin pathway, FSH, FSHR, macrophage, renal tubulointerstitial fibrosis

## Abstract

Estrogen withdrawal in aging women contributes to the progression of chronic kidney disease (CKD). However, the effect of high circulating follicle‐stimulating hormone (FSH) levels on renal dysfunction remains unknown. In this study, blood samples from 3,055 postmenopausal women were collected and tested, which showed that there was a strong negative correlation between eGFR and FSH levels (*p* < 0.001), independent of LH, testosterone, and estradiol. Functional FSHR was detected in renal tubular epithelial cells. In vivo, high circulating FSH levels promoted a phenotype of tubulointerstitial fibrosis, characterized by increases in 24‐hr urine protein/creatinine ratio, serum Cr, serum BUN, and ECM deposition. Similar results obtained from cultured HK‐2 cells showed that FSH increased the transcriptional and protein expression of profibrotic mediators (collagen IV, fibronectin, and PAI‐1). This promotion of fibrosis by FSH occurred through the activation of AKT/GSK‐3β/β‐catenin pathway, which could be attenuated by silencing FSHR by siRNA or by LY294002 or MK2206. In addition, FSH‐stimulated HK‐2 cells secreted IL‐8, which promoted macrophage migration to exacerbate tubulointerstitial fibrosis. These results revealed a previously unknown effect of FSH on kidney injury, which may offer a critical insight into the development of CKD in aging postmenopausal women.

## 1 INTRODUCTION

Chronic kidney disease is becoming a major global public health problem and greatly increases the risk of all‐cause mortality and cardiovascular mortality in aging women (Zhang et al., 2012). However, the underlying mechanisms remain largely unknown. Epidemiological studies showed that the incidence of CKD increased substantially after menopause (Holley, 2004). In the United States, 42% of the entire dialysis population are women, most of whom are in the postmenopausal period (Saran et al., 2018). It is widely reported that endogenous sex hormones, originally considered to act on the reproductive system, contribute to the development of kidney diseases, but their overall impacts on renal function still remain controversial. Quite a few studies held the view that female sex hormones were overall protective in terms of renal diseases because accelerated progression of CKD in males was more frequent than females (Kang & Miller, 2002; Saran et al., 2015; Silbiger & Neugarten, 1995). However, the sex differences in this renoprotection seem to diminish after menopause, when stratified by age (Nitsch et al., 2013). Furthermore, hypothalamic–pituitary–gonadal axis dysfunction ultimately brings about a decline in the estrogen level and was considered to be the key factor for the increased incidence of CKD, while estrogen supplementation alone failed to fully reverse renal dysfunction (Holley, 2004; Pietrzak et al., 2006). Together, these findings suggest that some sex hormones may play roles, which have not yet been investigated, in the pathophysiology of postmenopausal CKD.

A substantial elevation in the serum follicle‐stimulating hormone level, although likely to compensate for estrogen withdrawal, has been closely involved in the adverse effects of menopause on many nongonadal tissues, including bone (Sponton & Kajimura, 2017), liver (Song et al., 2016), adipose tissue (Liu et al., 2015), umbilical vascular endothelial cell (Siraj et al., 2013), and biliary epithelium (Onori et al., 2013). Functional follicle‐stimulating hormone receptors (FSHRs) were also reported to exist in these extragonadal tissues to regulate bone, glucose, and lipid metabolism (Qi et al., 2018; Sun et al., 2006). In addition, the abundance of FSHR in patients with primary renal carcinoma was nearly comparable to that in testicular cancer, in which the surge was different from the physiologically low FSHR expression in the kidneys (Fagerberg et al., 2014). FSHR can serve as an effective tumor vasculature marker, whose elevation is strongly related to angiogenesis in renal metastatic tumors (Siraj, Pichon, Radu, & Ghinea, 2012). However, to date, epidemiological and experimental studies exploring the potential association between FSH and CKD are not available, and no information exists about a functional contribution of FSH to kidney disease. Thus, the aim of this study was to investigate the hypothesis that FSH participates in the development of renal impairment during the postmenopausal period.

## 2 RESULTS

### 2.1 FSH is associated with eGFR, decreased eGFR, and CKD in postmenopausal women

**Figure 1:**
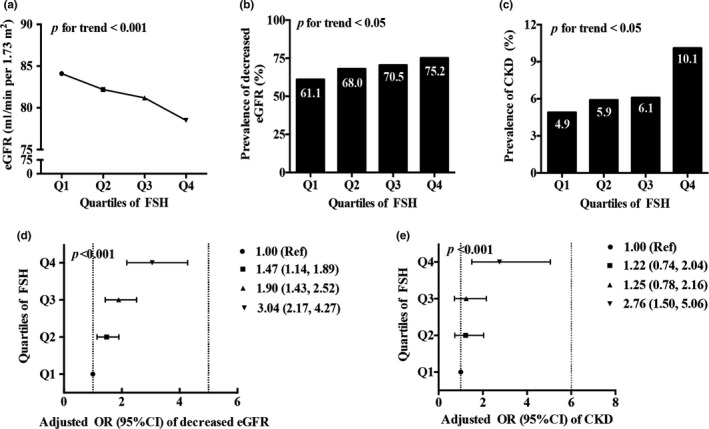
FSH is associated with eGFR, decreased eGFR, and CKD in postmenopausal women. (a) eGFR level in each quartile of FSH in postmenopausal women. (b, c) The prevalence of decreased eGFR and CKD in quartiles of FSH, respectively. (d, e) ORs of decreased eGFR and CKD by FSH quartile, respectively. Data are represented as the mean ± *SEM*. *p < 0.05

To confirm our hypothesis that FSH participates in the development of renal impairment during the postmenopausal period, we selected 3,055 postmenopausal women from 10,441 participants based on a cross‐sectional study called “SPECT‐China” (Figure S1) and then analyzed serum renal function indexes and sex hormone levels to explore whether FSH is related to renal function. The serum FSH level was first divided by quartile, and its quartile ranges were ≤47.30, 47.31–61.10, 61.11–78.10, and ≥78.11 IU/L (Table S1). With the increase in the FSH quartiles, eGFR levels decreased from 84.1 to 78.5 ml/min per 1.73 m^2^ (*p* for trend <0.001, Figure 1a). Consistently, the prevalence of decreased eGFR and CKD gradually increased across FSH quartiles (*p* for trend <0.001, Figure 1b,c). After adjusting for age, total testosterone (T), E2, LH, current smoker status, urban/rural residence, high/low economic status, BMI, dyslipidemia, diabetes, and hypertension, the odds risks (ORs) of decreased eGFR (OR = 3.04, 95% CI: 2.17–4.27) and CKD (OR = 2.76, 95% CI: 1.50–5.06) in the highest quartile of FSH increased approximately threefold compared with that in the lowest quartile (*p* for trend <0.001, Figure 1d,e), respectively. These clinical associations caused us to further examine the effect of FSH on renal fibrosis development.

### 2.2 Expression of FSHR in kidney and high FSH exposure exacerbated renal dysfunction

**Figure 2:**
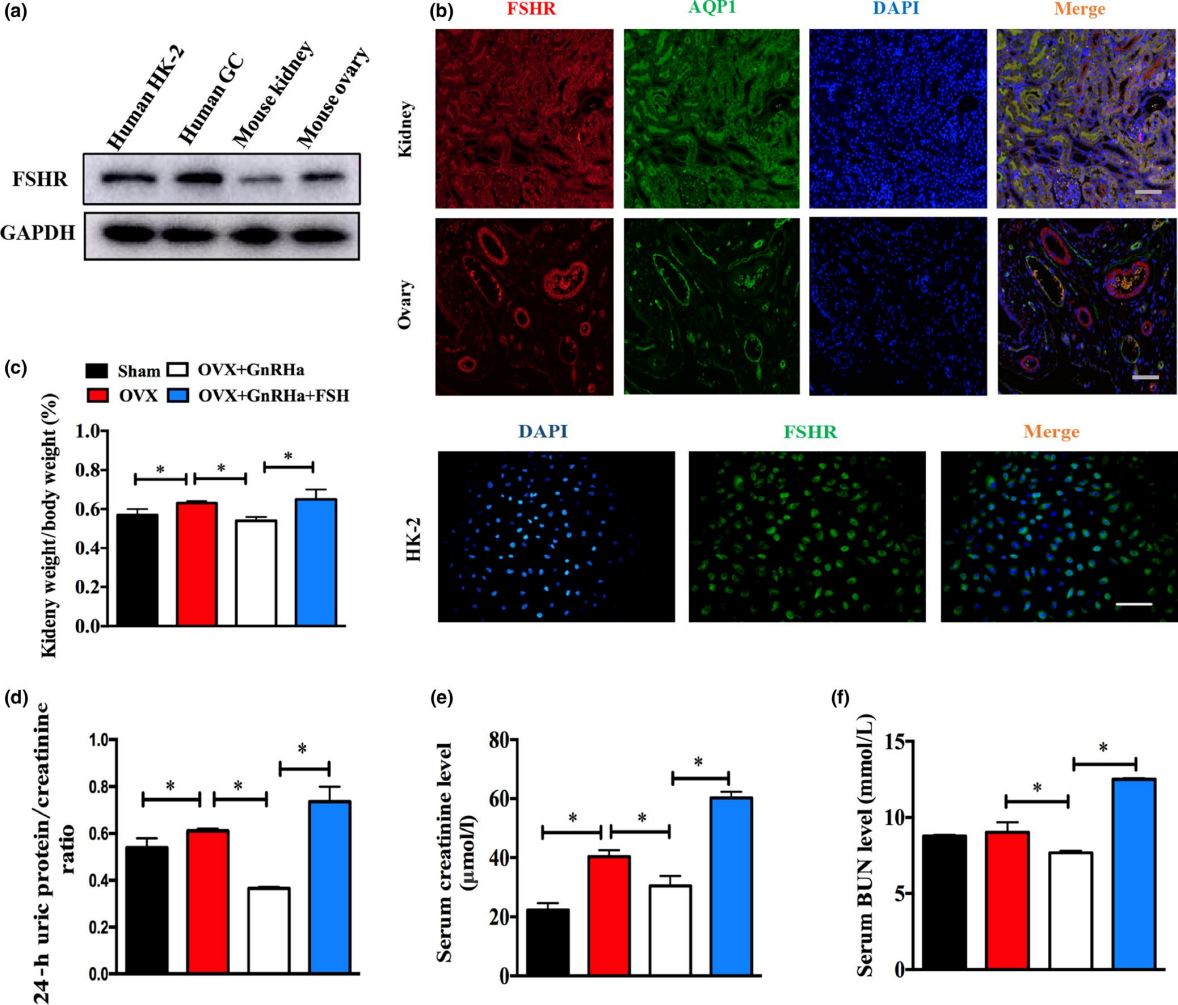
Expression of FSHR in normal mouse kidneys and high FSH exposure exacerbated renal dysfunction. (a) FSHR protein expression in normal mouse kidneys and HK‐2 cells. Human GC cells and mouse ovaries served as positive controls. (b) Localization of FSHR (red) in normal mouse kidneys, mouse ovaries, and HK‐2 cells. AQP1 (green) was mainly expressed in renal proximal tubules and vascular endothelium. Nuclei (blue) were stained with DAPI. Scale bar, 25 μm. (c) Kidney weight/body weight, (d) 24‐hr urine protein/creatinine ratio, (e) serum Cr, and (f) serum BUN in four groups (*n* = 6 or 7). Protein expression levels were normalized with GAPDH. Data were represented as the mean ± *SEM*. AQP1, aquaporin 1; GC, granulosa cell. *p < 0.05

To understand the roles of FSH in kidney disease, we first examined the expression of FSHR in kidney samples from normal female mice. As shown in Figure 2a, the expected band of FSHR sized at 78 kDa was detected in mouse kidneys, as well as FSHR‐rich human granulosa cells (GCs) and mouse ovaries. The protein localization of FSHR was further confirmed by immunofluorescence staining, showing that FSHR was highly abundant in mouse renal tubular epithelium and HK‐2 cells (Figure 2b). Thus, we speculated that renal proximal epithelium cells were likely to be the key candidate sites where FSH exerted an effect on the kidney. In addition, we attempted to investigate whether the FSHR is a functional FSHR when exposed to FSH treatment in vitro, and found that FSHR expression generally increased in a dose‐dependent manner in HK‐2 cells treated with FSH (Figure S2). These results suggest that FSHR may mediate FSH‐related kidney changes.

To confirm whether high FSH levels exert impacts on renal function in mice, we performed ovariectomy surgery to mimic postmenopausal hormone changes (Figure S3A). As expected, OVX mice had declined levels of E2 as well as increased levels of LH and FSH (Figure S3C,D), and obvious elevations in kidney weight/body weight, 24‐hr urine protein/creatinine, serum creatinine, and serum urea nitrogen (Figure 2c–f), but no significant changes in body weight (Figure S3B) were observed. Ovariotomy‐induced renal dysfunction was improved by daily administration of GnRHa, a gonadotropin‐releasing hormone agonist that can suppress pituitary‐derived FSH and LH release. Additional FSH injected into mice was used to explore the singular effect of FSH on renal dysfunction as shown in Figure 2c–f described, and FSH supplementation in the absence of LH led to a marked aggravation in renal dysfunction.

### 2.3 High FSH impaired kidney function through enhanced tubulointerstitial fibrosis

**Figure 3:**
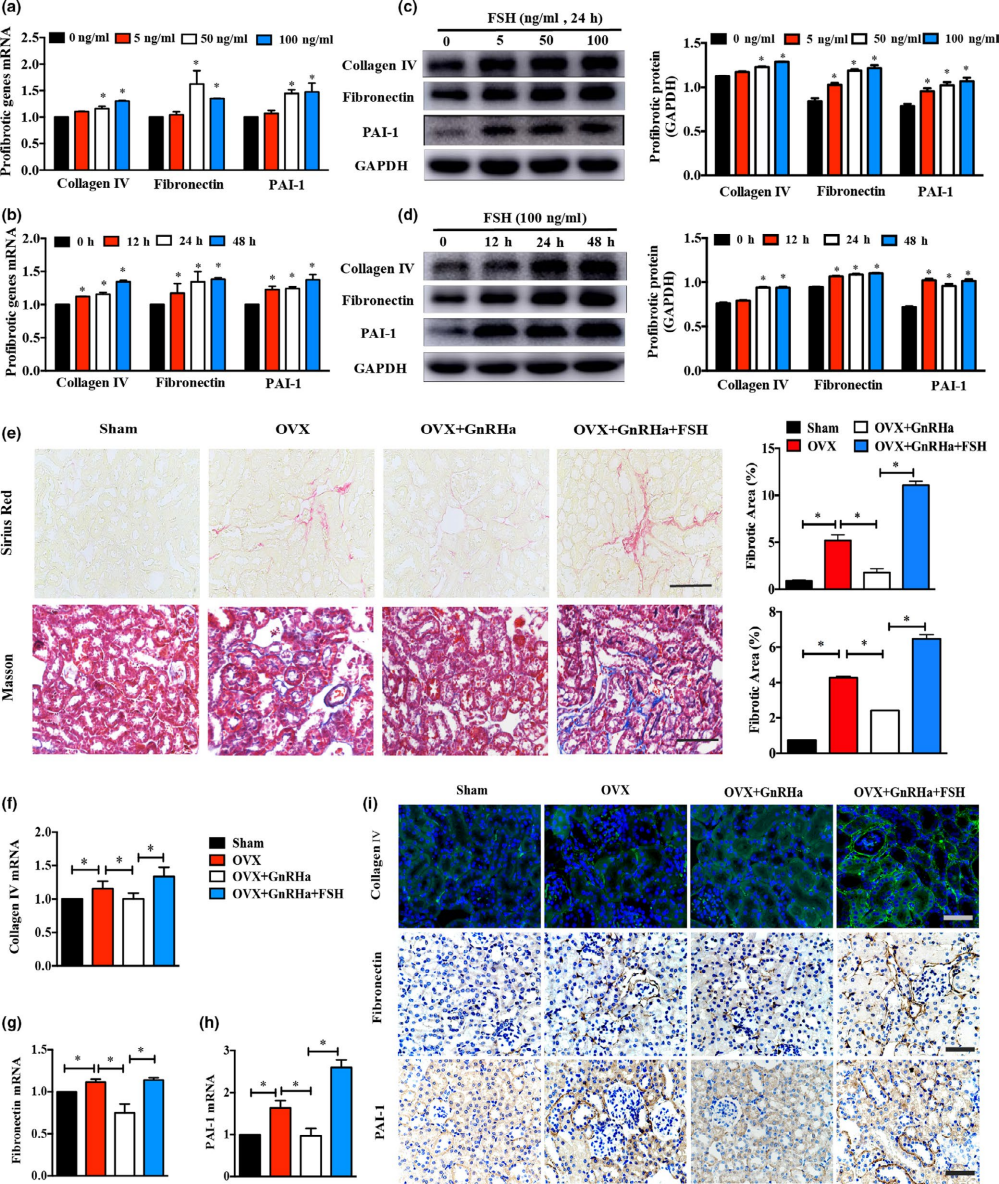
High FSH impaired the kidneys through enhanced tubulointerstitial fibrosis. (a–d) Collagen IV, fibronectin, and PAI‐1 transcriptional and protein expressions in HK‐2 cells treated with different doses of FSH for 24 hr or treated with FSH (100 ng/ml) for different times as indicated. (e) Collagen deposition was assessed by Masson and Sirius Red staining in mouse kidney samples from four groups. Scale bar, 25 μm. (f–h) RT‐PCR analyses of profibrotic markers in kidney samples from different experimental groups. (i) Immunostaining images of kidney sections from four groups for collagen IV (green), fibronectin (brown), and PAI‐1 (brown). Scale bar, 25 μm. Transcriptional and protein expression levels were normalized with β‐actin or GAPDH, respectively. Data were represented as the mean ± *SEM*. *p < 0.05

Given that functional FSHR was highly elevated in renal tubular epithelium, we mainly focused on HK‐2 cells to further understand how FSH exacerbated renal dysfunction. In vitro, the transcriptional and protein expression of several fibrotic genes (collagen IV, fibronectin, and PAI‐1) significantly increased in a time‐ and dose‐dependent manner in HK‐2 cells (Figure 3a–d), while we did not observe an obvious difference in cell viability (Figure S4), which led us to speculate that renal tubulointerstitial fibrosis, rather than apoptosis, might be responsible for FSH‐induced renal dysfunction. Renal fibrosis is characterized by excessive fibrous connective tissues, such as collagen and fibronectin in or around lesions. In vivo, renal Sirius Red staining and Masson staining were performed which showed that high circulating FSH groups (OVX vs. OVX + GnRHa + FSH mice) have more interstitial collagen deposition, whose positive areas increased approximately threefold compared to low circulating FSH group (OVX + GnRHa mice; Figure 3e). We further validated the mRNA and protein expression levels of profibrotic factors by quantitative RT‐PCR and Western blot and found that collagen IV, fibronectin, and PAI‐1 expression levels in the high circulating FSH groups were notably upregulated compared to those in low circulating FSH group (Figure 3f–h). These identical alterations were also observed in the IHC and IF results (Figure 3i).

### 2.4 The AKT/GSK‐3β/β‐catenin signaling pathway was essential for FSHR‐mediated interstitial fibrosis

**Figure 4:**
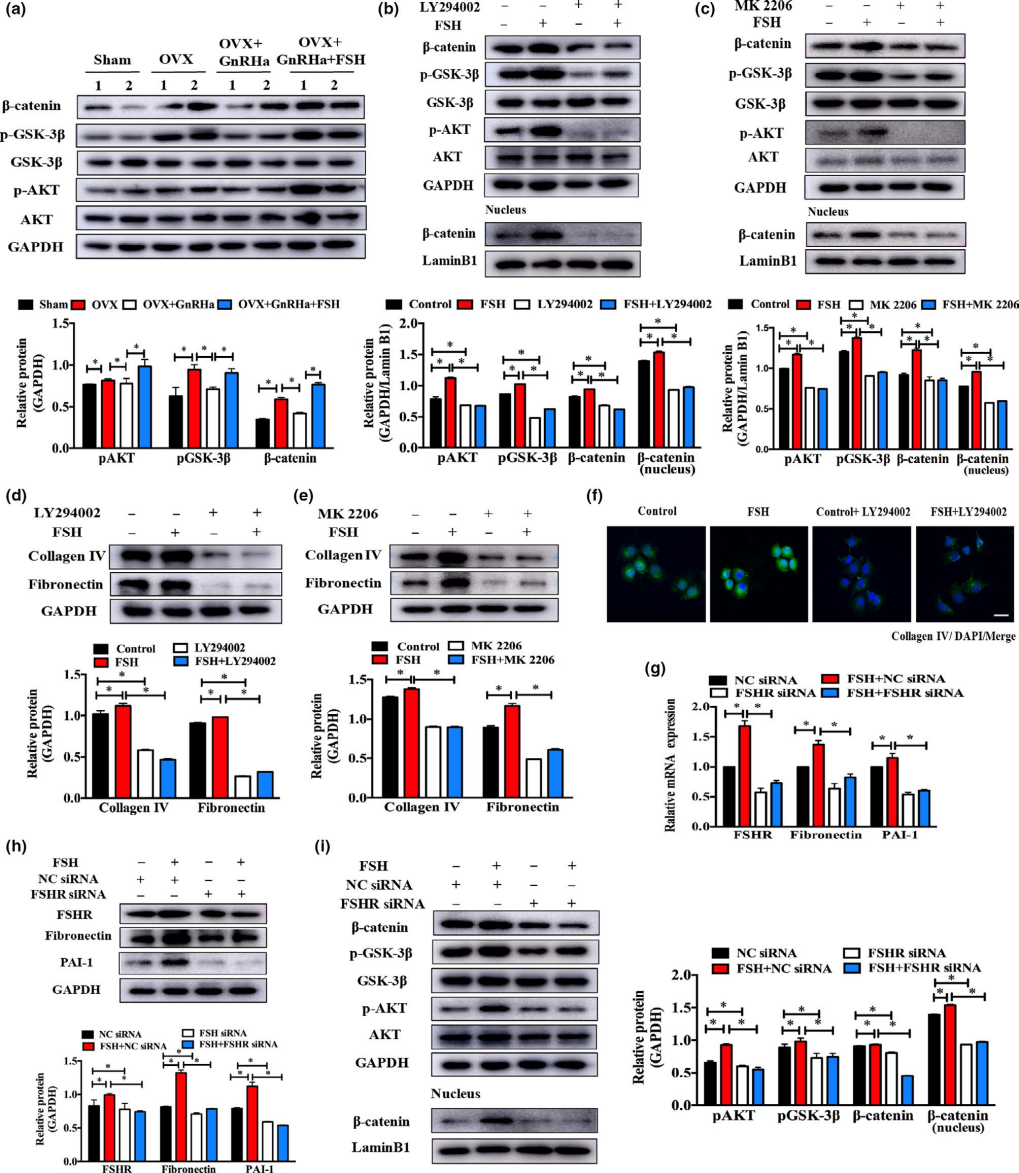
AKT/GSK‐3β/β‐catenin signaling pathway was essential for FSHR‐mediated interstitial fibrosis. (a) Protein analyses for pAKT (Thr308), pGSK‐3β, and β‐catenin as well as corresponding total protein in mouse kidney samples from different experimental groups. (b, c) Protein analyses for pAKT (Thr308), pGSK‐3β, and β‐catenin as well as corresponding total protein in HK‐2 cells treated with FSH (100 ng/ml) for 15 min in the presence or absence of LY294002 (50 μM, 2 hr) or MK 2206 (10 μM, 2 hr). (d, e) Protein expressions of collagen IV and fibronectin in HK‐2 cells exposed to 100 ng/ml FSH for 24 hr in the presence or absence of LY294002 (50 μM, 24 hr) or MK 2206 (10 μM, 24 hr). Scale bar, 25 μm. (f) Immunostaining images of HK‐2 cells for collagen IV (green). Scale bar, 25 μm. (g, h) Transcriptional and protein levels of FSHR, fibronectin, and PAI‐1 in FSH‐treated HK‐2 cells for 24 hr with or without FSHR siRNA. (i) pAKT (Thr308) and total AKT, pGSK‐3β, GSK‐3β, and β‐catenin in HK‐2 cells treated with FSH at 24 hr in the absence or presence of FSHR siRNA. GAPDH and lamin B1 were used as internal markers for total and nuclear protein, respectively. Quantitative analyses for FSHR by densitometry with ImageJ. *p < 0.05

Since high circulating FSH levels were found to cause renal ECM (extracellular matrix) deposition and interstitial fibrosis, we mainly concentrated on the signaling pathways that might be involved in renal fibrosis reported in recent literature. We first examined the protein changes of pAKT (Thr308), AKT, pGSK‐3β (Ser9), and GSK‐3β, and we found that phosphorylation of AKT (Thr308) and GSK‐3β (Ser9) was increased in OVX and OVX + GnRHa + FSH mice compared with that in OVX + GnRHa mice (Figure 4a), respectively. No significant changes in the corresponding total proteins were observed. The results in HK‐2 cells treated with FSH were consistent with those in kidney samples (Figure S5). Enhanced phosphorylation of GSK‐3β (Ser9) led to its own inactivation, causing β‐catenin accumulation. As expected, the protein expression of β‐catenin, a downstream signaling molecule, increased in both the cytoplasm and the nuclei of HK‐2 cells (Figure S5). To further examine whether the activation of AKT signaling is indispensable for FSH‐induced ECM accumulation, LY294002 and MK 2206, two common AKT pathway inhibitors, were added to HK‐2 cells. LY294002 or MK 2206 caused a marked inhibition of phospho‐AKT (Thr308), phospho‐GSK‐3β (Ser9) phosphorylation, and a reduction of the β‐catenin level (Figure 4b,c). Moreover, LY294002 or MK 2206 reversed the fibrotic phenotypes demonstrated by Western blot, which effectively decreased FSH‐stimulated protein levels of collagen IV and fibronectin in HK‐2 cells (Figure 4d,e). This was consistent with a reduced expression of collagen IV, as assessed by immunofluorescence (Figure 4f). These results indicated that FSH can enhance the downstream AKT signaling pathway to inactivate GSK‐3β, followed by β‐catenin accumulation and translocation into the nucleus to activate fibrosis‐related genes.

Given that FSH exerts its biological effect via binding to FSHR, we next attempted to silence FSHR function via the specific siRNA to explore whether FSH‐induced fibrogenic action was mediated by FSHR. Effective ablation of FSHR was obtained with an approximately twofold decline in the transcriptional and protein expression in HK‐2 cells (Figure 4g,h). FSHR knockdown significantly reduced FSH‐stimulated transcription and protein levels of PAI‐1 and fibronectin, suggesting that FSH‐mediated renal fibrosis is dependent on functional FSHR (Figure 4g,h). We next tested the effect of FSHR on FSH‐induced signaling transduction. Western blotting analyses revealed that FSHR ablation inhibited FSH‐stimulated phosphorylation of AKT and GSK‐3β, as well as β‐catenin accumulation and its nuclear transduction in HK‐2 cells (Figure 4i). These results indicated that FSHR is also required for FSH‐induced signaling transduction.

### 2.5 Macrophages exacerbated FSH‐mediated interstitial fibrosis

**Figure 5:**
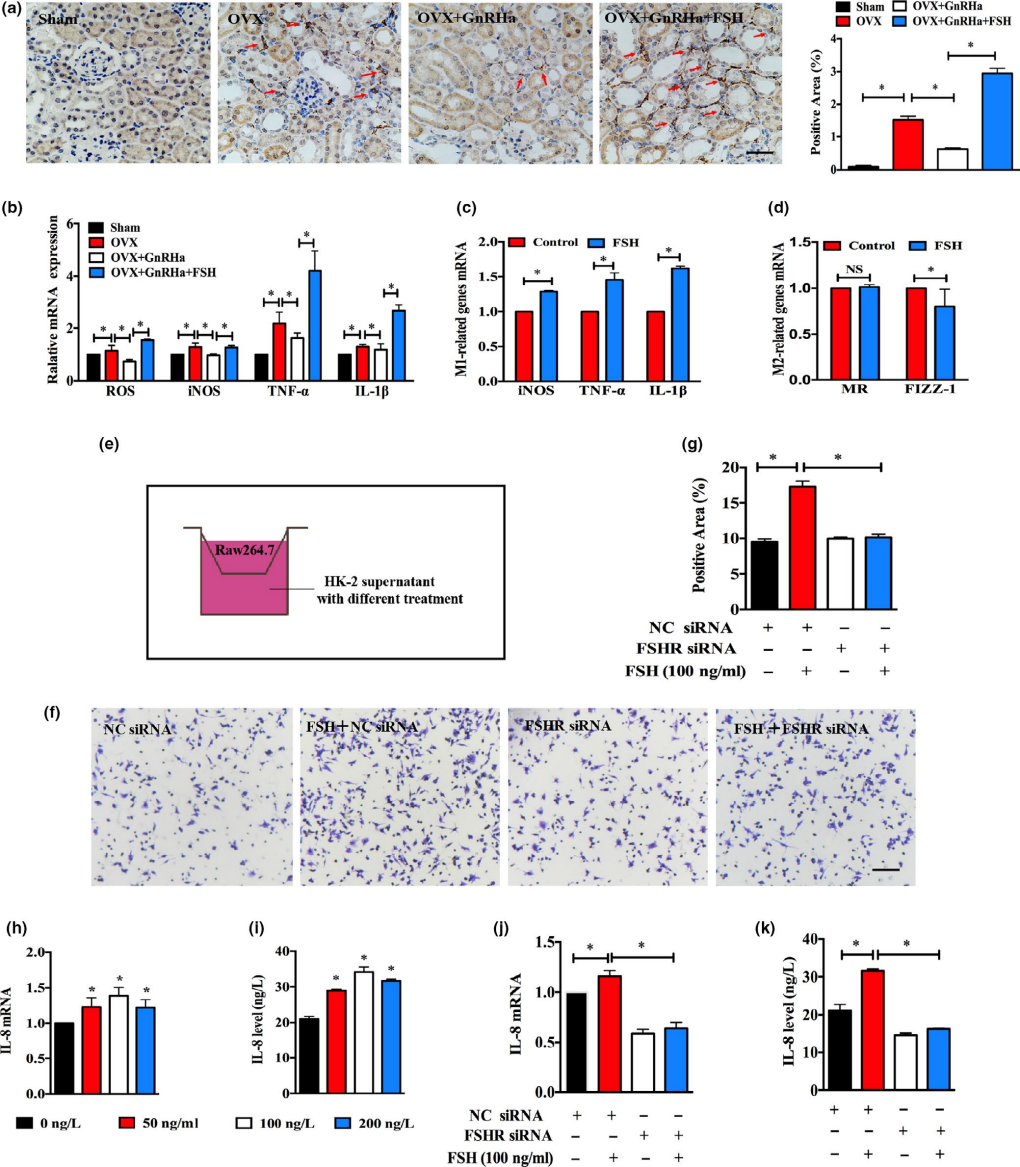
FSH contributed to the activation of macrophage and migration. (a) Representative images of macrophage biomarker, F4/80, in sham and ovariectomized kidneys from different experiment groups. Scale bar, 25 μm. (b) ROS, iNOS, TNF‐α, and IL‐1β transcriptional expressions in kidney samples from different groups. (c, d) Transcriptional levels of M1‐ or M2‐related genes in FSH‐treated Raw264.7 cells for 24 hr, respectively. (e) Diagram of the coculture system. (f) 1 × 10^6^/ml Raw264.7 cells' FSH‐treated HK‐2 supernatant with NC siRNA or FSHR siRNA. (g) Quantitative analyses of macrophage numbers. (h–k) IL‐8 gene expression and IL‐8 protein level in HK‐2 cells or supernatant treated with FSH at different concentrations (0–200 ng/ml) or with NC siRNA or FSHR siRNA. Transcriptional and protein expression levels are normalized to β‐actin or GAPDH or lamin B1. Scale bar, 25 μm. Quantitative analyses for FSHR by densitometry with ImageJ. Data were represented as the mean ± *SEM*. *p < 0.05

Damaged epithelial cells release a train of chemokines that can draw macrophages to the sites of injured tissue for inflammation. We observed that highly circulating FSH group had more macrophages as supported by IHC in kidney sections (Figure 5a). Several toxic factors (ROS and iNOS) and proinflammatory cytokines (TNF‐α and IL‐1β) secreted by innate macrophages were significantly increased in the high circulating FSH group compared to the low circulating FSH group, as assessed by RT‐PCR (Figure 5b). In vitro, macrophages exposed to FSH stimulation exhibited similar results, and these cytokine changes were also the markers of macrophage polarization from M0 to M1, indicating that FSH induced macrophages to change to a proinflammatory phenotype (Figure 5c,d). It is widely believed that damaged epithelial cell‐secreted chemokines draw macrophages to the sites of injured tissue; then, we performed a transwell assay and found that macrophage migration was indeed enhanced when cocultured with the FSH‐treated HK‐2 supernatant, and this promotion was attenuated by FSHR blockage (Figure 5e–g). IL‐8, a powerful chemotactic factor secreted by renal epithelial cells, markedly increased in patients with kidney diseases; therefore, we used RT‐PCR and ELISA to test the effect of FSH on IL‐8 levels in vitro. As expected, IL‐8 levels gradually increased, and the optimal stimulated concentration of FSH was likely to be at a dosage of 100 ng/ml (Figure 5h,i). Next, we silenced FSHR by FSHR siRNA based on this concentration, and the chemotactic influence of IL‐8 was significantly suppressed (Figure 5j,k). Together, these data indicate that macrophages exacerbated FSH‐mediated interstitial fibrosis.

## 3 DISCUSSION

Numerous epidemiological studies have documented that FSH sharply increased during the postmenopausal period in which CKD occurred rather rapidly (Burger et al., 1995; Saran et al., 2018). However, no clinical correlation between renal function and serum FSH levels has been explored. Here, we analyzed data from 3,055 postmenopausal samples from a large cross‐sectional survey that enrolled more than 10,000 Chinese individuals, showing that high circulating FSH level increased the risks of decreased eGFR and CKD in the postmenopausal female population, and this positive correlation was independent of age, total T, estradiol, current smoker status, etc. Based on large‐scale clinical data analysis, we attempted to investigate whether FSH participated in the progression of kidney disease in experimental study.

Postmenopausal status is characterized by high levels of FSH and LH, as well as low levels of E2, making it difficult to explore the precise role of FSH in renal function in the OVX mouse model. To diminish the potential effect of these two parameters, we used GnRHa‐treated ovariectomized mice administered with recombinant FSH in accordance with several studies (Li, Chen, et al., 2017; Song et al., 2016). Given that kidney injury is a relatively chronic process, we increased the dose of FSH from 0.15 IU to 0.3 IU and lengthened intervention time to 6 weeks, which was different from Liu's study on fat accumulation. Consequently, rising serum FSH and falling serum LH and E2 were all observed in OVX + GnRHa + FSH mice. Under these conditions, OVX + GnRHa + FSH mice had higher levels of 24‐hr uric protein/creatinine, serum Cr, and serum BUN and presented a more obvious phenotype of ECM deposition by histopathology, suggesting that FSH was responsible for renal dysfunction and tubulointerstitial fibrosis. These results also indirectly eliminated the effect of GnRHa on kidney injury as Gandaglia's findings reported (Gandaglia et al., 2014). Interstitial fibrosis exhibited identical manifestations in all progressive forms of chronic kidney disease, although glomerular lesions played a specific role in the pathogenesis of renal disease (Zeisberg & Neilson, 2010). Tubulointerstitial fibrosis seen in ovariectomized mice might be partly attributed to abundant expression of FSHR in the renal tubular epithelium. The transition of tubular epithelial cells (TECs) from an epithelial phenotype to mesenchymal feature contributed to tubulointerstitial fibrosis (Li, Li, Hao, Liu, & Duan, 2017). Consistent with these findings, FSH treatment significantly increased transcriptional and protein expression of fibrogenic cytokines (PAI‐1, fibronectin, and collagen IV) in vitro and in vivo.

Follicle‐stimulating hormone receptor is a member of the G protein‐coupled receptor that is considered as a driver of the activation of multiple signaling cascades. Emerging evidence from studies suggests that an altered PI3K/AKT signaling pathway is highly linked to the pathogenesis of renal fibrosis. FSH stimulated the FSHR‐mediated PI3K pathway, leading to activation of AKT and downstream targets in GCs (Hunzicker‐Dunn et al., 2012). A report by Cheryl et al. demonstrated that FSHR interacts with the adaptor protein APPL1 via the PI3K/AKT pathway to promote cell survival in HEK 293 cells in response to FSH treatment (Nechamen et al., 2004). As shown in our findings, FSH‐stimulated rapid AKT (Thr308) phosphorylation was observed in HK‐2 cells, and the inhibition of PI3K/AKT signaling with LY294002 or MK 2206, or the silencing of FSHR by siRNA, significantly reduced this activation. GSK‐3β is believed to exert an anti‐fibrotic effect in multiple organ systems (Gong, Rifai, Ge, Chen, & Dworkin, 2008). The enhancement in AKT phosphorylation activates GSK‐3β by phosphorylation at Ser9, thus leading to changes in activity of GSK‐3β. Further, GSK‐3β targets the β‐catenin complex for ubiquitination and proteasomal degradation, and the disintegration of this complex contributes to β‐catenin accumulation and nuclear translocation, thus triggering fibroblast activation and fibrogenesis (Hwang, Seo, & Ha, 2009). FSH promoted the phosphorylation of GSK‐3β and β‐catenin translocation into the nucleus, and LY294002 or MK 2206, or FSHR siRNA powerfully blocked GSK‐3β/ β‐catenin signaling and lessened the expression of profibrotic proteins mediated by FSH, such as fibronectin and collagen IV.

Numerous evidence has suggested that macrophages have crucial roles in the progression of fibrosis (Wynn, 2011). Macrophages have 2 distinct subtypes participating in this process: classically activated (M1) and alternatively activated (M2) macrophages. M1 macrophages generally infiltrated and presented profibrotic activity by releasing toxic factors that can aggravate tissue injury, such as ROS, iNOS, TNF‐α, and IL‐1β (Wynn & Barron, 2010). Indeed, we found that FSH triggered classical macrophage activation in vitro, which was characterized by increased transcriptional levels of proinflammatory factors. Interestingly, damaged epithelial cells and endothelial cells per se secrete a series of chemotactic cytokines that can recruit macrophages to the site of diseased tissue (Wynn & Ramalingam, 2012). IL‐8 is well believed to be a key chemotactic cytokine, which is involved in the recruitment of monocyte (Meniailo et al., 2018). Profibrotic mediators such as TGF‐β induced proximal tubular cells to secrete IL‐8, and its production was markedly enhanced in urine proteins obtained from patients with tubular injury (Huang, Wen, Zhou, & Yu, 2008; Qi et al., 2006; Singhto & Thongboonkerd, 2018). In our results, FSH‐treated HK‐2 cells resulted in the production of chemokine IL‐8, which, in turn, induced FSHR‐mediated macrophage migration.

In conclusion, we have identified that FSH promoted renal dysfunction and tubulointerstitial fibrosis via the AKT/GSK‐3β/β‐catenin pathway in HK‐2 cells. FSH also activated macrophage polarization and recruited macrophages to exacerbate renal injury. Our results revealed a previously unknown function of FSH on nongonadal tissues of the kidney, which will enhance the understanding of the pathophysiology of CKD and will benefit millions of aging women.

## 4 MATERIALS AND METHODS

### 4.1 Clinical data

The data are from 10,441 participants of SPECT‐China, a cross‐sectional survey in East China (ChiCTR‐ECS‐14005052, www.chictr.org.cn). Recruitment and enrollment have been previously described in detail (Wang, Cheng, et al., 2017; Wang et al., 2015; Wang, Wang, et al., 2017). A total of 3,226 postmenopausal women were selected. Postmenopausal women were defined as subjects who reported that they had stopped menstruating for a minimum of 12 months (*n* = 1,431), who were 55 years of age or older (*n* = 2,872), or who had previous hysterectomy or oophorectomy (*n* = 139). Exclusion criteria included missing values of FSH, luteinizing hormone (LH) or eGFR (*n* = 12), and FSH < 25.0 IU/L (per the 2011 Stages of Reproductive Aging Workshop +10 recommendation, late perimenopausal state is characterized as FSH level ≥ 25 IU/L; *n* = 159) (Harlow et al., 2012). Finally, 3,055 participants were included in the study, as shown in Figure S1.

The estimated glomerular filtration rate (eGFR) was calculated according to the Chronic Kidney Disease Epidemiology Collaboration (CKD‐EPI) equation (Levey et al., 2009). eGFR < 90 ml/min per 1.73 m^2^ or 60 ml/min per 1.73  m^2^ was defined as decreased eGFR or CKD, respectively. The study protocol was approved by the Ethics Committee of Shanghai Ninth People's Hospital, Shanghai Jiao Tong University School of Medicine. All procedures followed were in accordance with the ethical standards of the responsible committee on human experimentation (institutional and national) and with the Helsinki Declaration of 1975, as revised in 2008. Informed consent was obtained from all patients included in the study.

### 4.2 Animals

Female mice were randomly assigned into the following four groups: sham operation (Sham, *n* = 6), bilateral ovariectomy (OVX, *n* = 7), OVX + GnRHa (*n* = 7), and OVX + GnRHa + FSH (*n* = 8). At 8 weeks, all mice were subjected to sham operation or bilateral ovariectomy with a 1‐cm dorsal incision. Subsequently, the mice were allowed to recover for 1 week. Ovariectomy was performed to mimic the postmenopausal features of high FSH levels. Ovariectomized mice partly received an intraperitoneal injection of GnRHa triptorelin acetate (Beaufour‐Ipsen) 0.5 μg per day for 6 weeks to inhibit pituitary FSH and LH secretion. OVX + GnRHa + FSH mice were given an additional injection of recombinant FSH (Gonal‐f, Merck Serono) 0.3 IU daily for 4 weeks. Mice without GnRHa or FSH treatment were administered daily with saline and served as controls. All the procedures were conducted in accordance with the Animal Research Committee of Shanghai Ninth People's Hospital affiliated with Shanghai Jiao Tong University.

### 4.3 Biochemical measurement

Mice were housed individually in metabolic cages for 24 hr to collect urine samples. Serum creatinine (Cr), urea nitrogen (BUN), and other serum parameters were completed on Beckman Coulter AU 680 in the central laboratory. Serum FSH, LH, and E2 levels in mice were measured by radioimmunoassay at the North Biotechnology Institute (Beijing).

### 4.4 Cell culture and treatment

Human renal proximal tubular epithelial cell line (HK‐2) and mouse macrophage cells (RAW264.7) were obtained from the ATCC (American Type Culture Collection). HK‐2 cells were maintained in Dulbecco's modified Eagle's medium (DMEM)/Nutrition Mixture F‐12 supplemented with 10% fetal bovine serum (FBS, HyClone) and 1% penicillin–streptomycin (Gibco). HK‐2 cells were transfected with 50 nM FSHR siRNA using Lipofectamine 2000 (Invitrogen) to block the effect of FSHR. LY294002 (50 μM) or MK 2206 (10 μM) was added to inhibit PI3K/AKT signaling pathway. RAW264.7 cells were cultured in DMEM medium with 10% FBS and 1% penicillin–streptomycin and treated with FSH (100 ng/ml) for 24 hr. Cells were collected for gene and protein analyses.

### 4.5 Quantitative real‐time PCR

Total RNA was extracted from cells and kidney tissues using TRIzol reagent (Invitrogen) according to the manufacturer's protocol. Real‐time quantitative PCR was performed on an ABI Machine with a two‐step method, and β‐actin was served as an endogenous control. The relative mRNA expression levels of genes were calculated using the 2^−∆∆Ct^ method as previously described. Each RNA sample was repeated independently at least three times.

### 4.6 Western blot analysis

Cells and kidney proteins were extracted as previously described (Ning et al., 2017). Primary antibodies were as follows: anti‐FSHR (1:1,000; Abcam), anti‐PAI (1:1,000; Abcam), anti‐collagen IV (1:1,000; Abcam), anti‐fibronectin (1:1,000; Abcam), anti‐pAKT (Thr308, 1:1,000; CST), anti‐AKT (1:1,000; CST), anti‐pGSK‐3β (1:1,000, CST), anti‐GSK‐3β (1:1,000, CST), β‐catenin (1:1,000; CST), anti‐lamin B1 (1:2,500; Proteintech), and anti‐GAPDH (1:5,000; Sigma). Relative densitometry was calculated by the ImageJ software.

### 4.7 Morphological analyses and immunochemistry

Paraformaldehyde‐fixed, paraffin‐embedded mouse kidney sections were used for Masson's trichrome staining and Sirius Red staining. The fibrotic area was quantified by the ImageJ software. The corresponding values were expressed as a percentage of the entire area. For immunochemistry staining, paraffin sections were subjected to further dewaxing, rehydration, and antigen retrieval. Subsequently, slides were stained with PAI‐1 (1:100; Abcam), fibronectin (1:100; Abcam), and F4/80 (1:100; Abcam) antibodies. Images were captured using a Nikon microscope.

### 4.8 Immunofluorescence staining

Histopathologic slides and cultured HK‐2 cells were incubated with primary antibodies FSHR (1:100; Abcam), type IV collagen (1:100; Abcam), AQP1 (1:100; Abcam), and secondary antibodies including Alexa Fluor 488 and Alexa Fluor 594. Nuclei were counterstained with 4′‐6‐diamidino‐2‐phenylindole (DAPI). Images were captured using a Nikon microscope.

### 4.9 ELISA for IL‐8

HK‐2 cells were cultured for 24 hr with FSH or NC siRNA or FSHR siRNA, and their supernatants were collected. The levels of IL‐8 from HK‐2 supernatant were measured by a commercially available human IL‐8 assay kit according to the manufacturer's instructions.

### 4.10 Coculture and transwell assay

HK‐2 cells were transfected with NC siRNA or FSHR siRNA using Lipofectamine 2000 or IL‐8‐neutralizing antibody or treated with FSH for 24 hr, followed by supernatant collection. Subsequently, 2 × 10^5^/well macrophages suspended in FBS‐free DMEM/F12 medium were seeded into the upper chamber and HK‐2 cells supernatants were added in the lower chamber. This coculture system was incubated for 24 hr, which attracted macrophages to migrate toward the underside of the insert membrane. The migrated cells were fixed and stained with 0.1% crystal violet solution. Images were captured using a Nikon microscope.

### 4.11 Statistical analysis

Data analyses were performed using IBM SPSS Statistics, version 22 (IBM Corporation). The associations of FSH quartiles with decreased eGFR and CKD (categorical variable) were assessed by binary logistic regression. The model was adjusted for age, total testosterone (T), E2, LH, current smoker status, urban/rural residence, high/low economic status, BMI, dyslipidemia, diabetes, and hypertension. Two‐way analysis of variance (ANOVA) followed by Fisher's PLSD post hoc test was used to compare significant differences between groups. All analyses were two‐sided. A *p* value <0.05 indicated significance. Error bars in histograms indicate the standard error of the mean (*SEM*).

## CONFLICT OF INTEREST

None declared.

## AUTHOR CONTRIBUTIONS

K.Z., N.W., and L.Y.L. designed the study; K.Z. performed and drafted the experiment; N.W. performed clinical data analyses; L.K., F.X., and N.W. revised the manuscript; Y.C., W.Z., C.W., and H.Z. gave experimental support; Y.L. supervised the entire project; and all authors approved the final version of the manuscript.
