# Supplementary figures and images for: Follicle‐stimulating hormone promotes renal tubulointerstitial fibrosis in aging women via the AKT/GSK‐3β/β‐catenin pathway
Source: Aging Cell. 2019 Jun 26;18(5):e12997. doi: 10.1111/acel.12997 (PMC6718534; doi:10.1111/acel.12997)

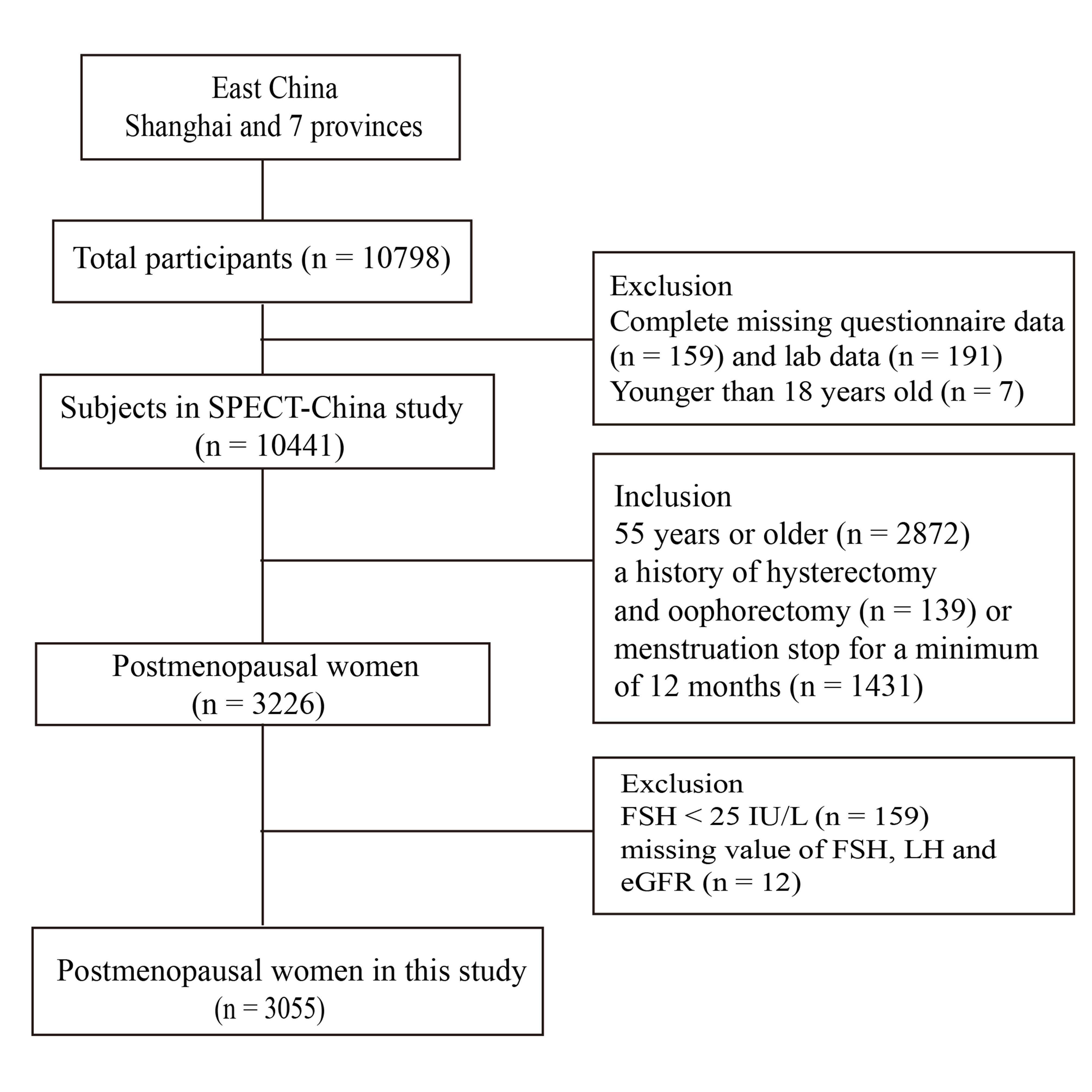

Supplement: Supplementary file 1 [file ACEL-18-e12997-s001.tif]

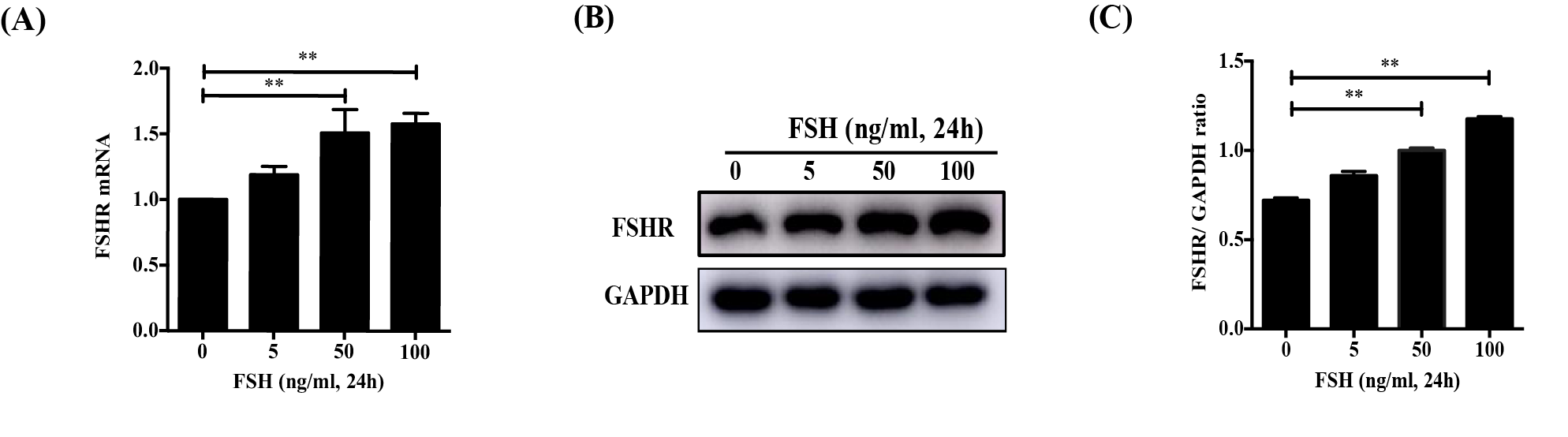

Supplement: Supplementary file 2 [file ACEL-18-e12997-s002.tif]

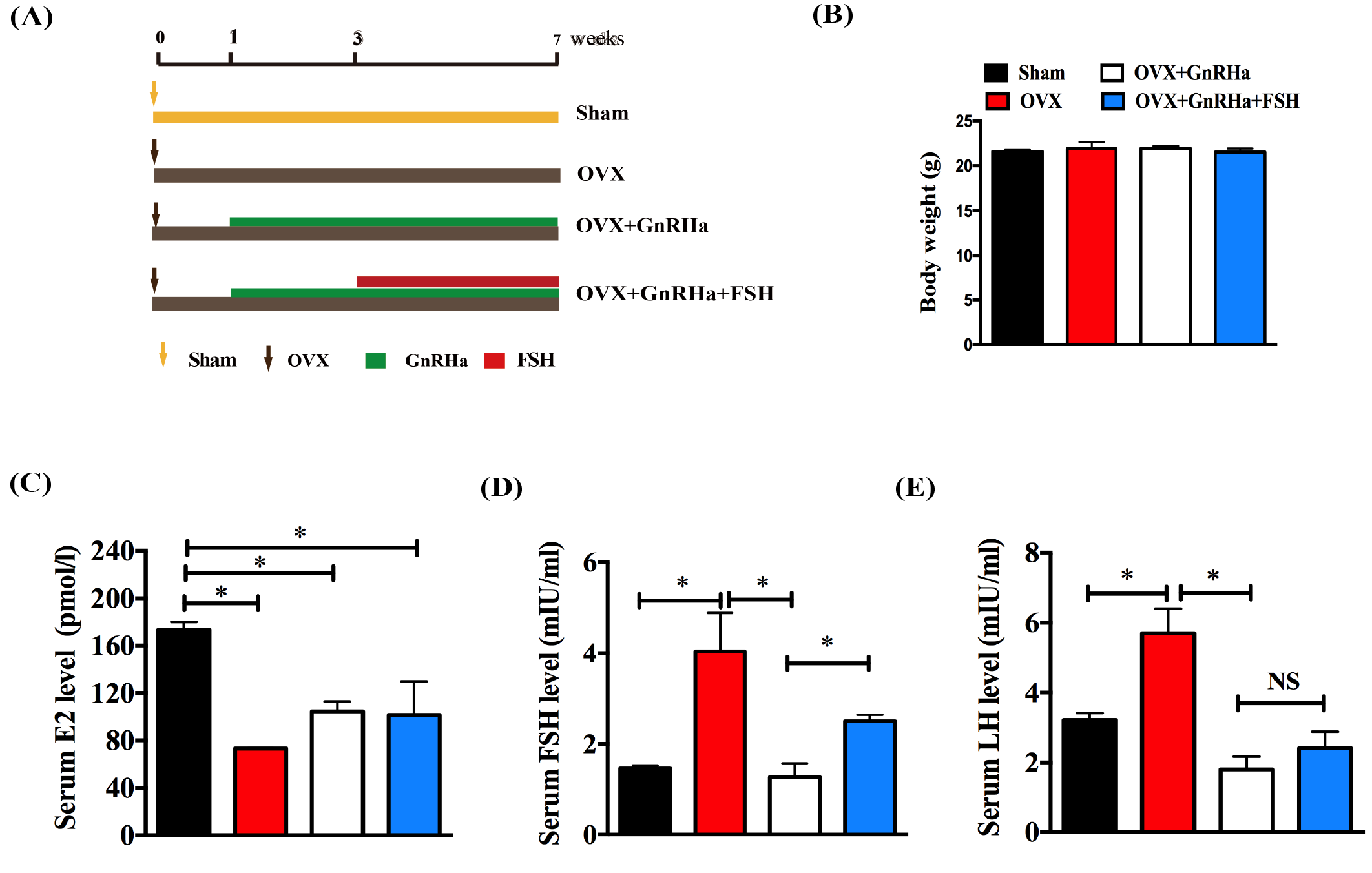

Supplement: Supplementary file 3 [file ACEL-18-e12997-s003.tif]

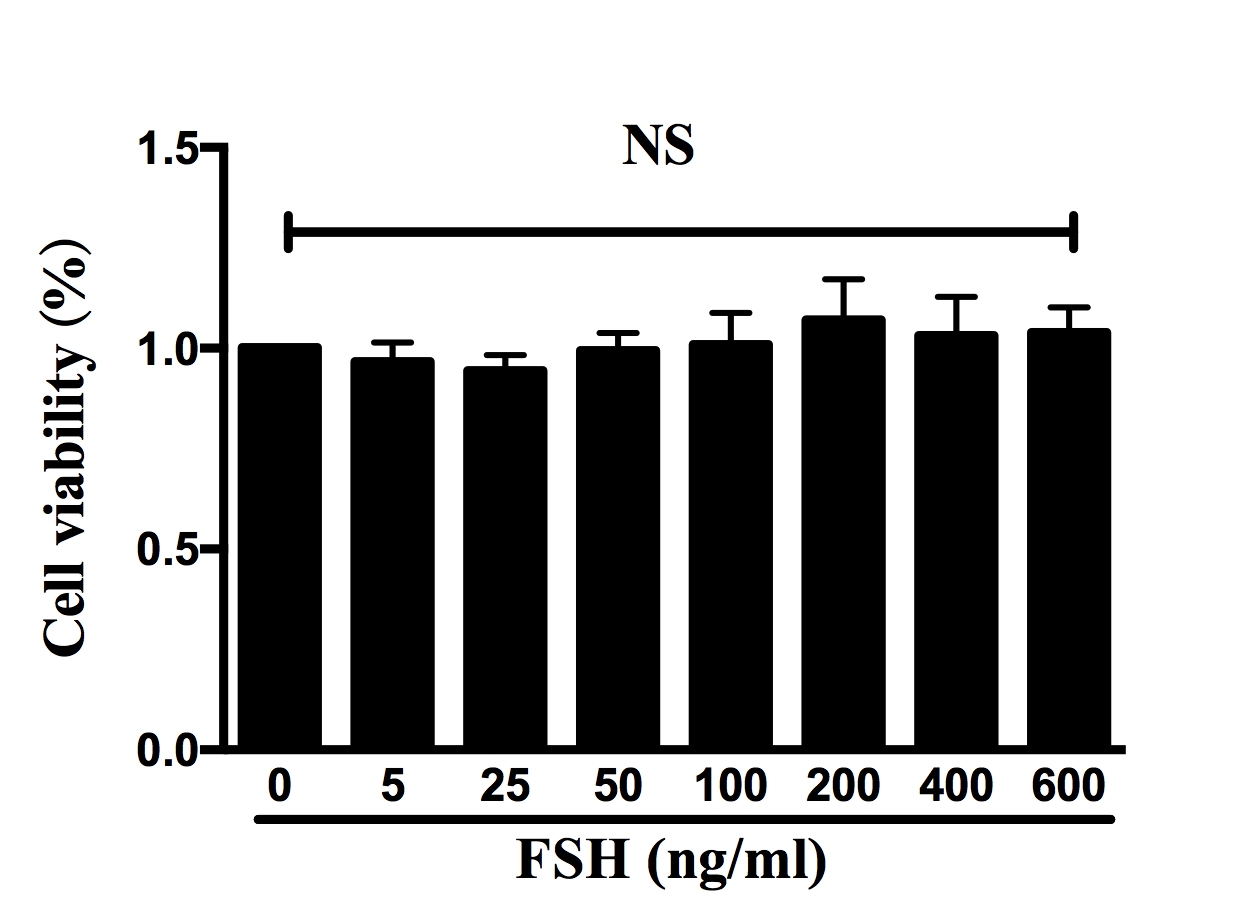

Supplement: Supplementary file 4 [file ACEL-18-e12997-s004.tif]

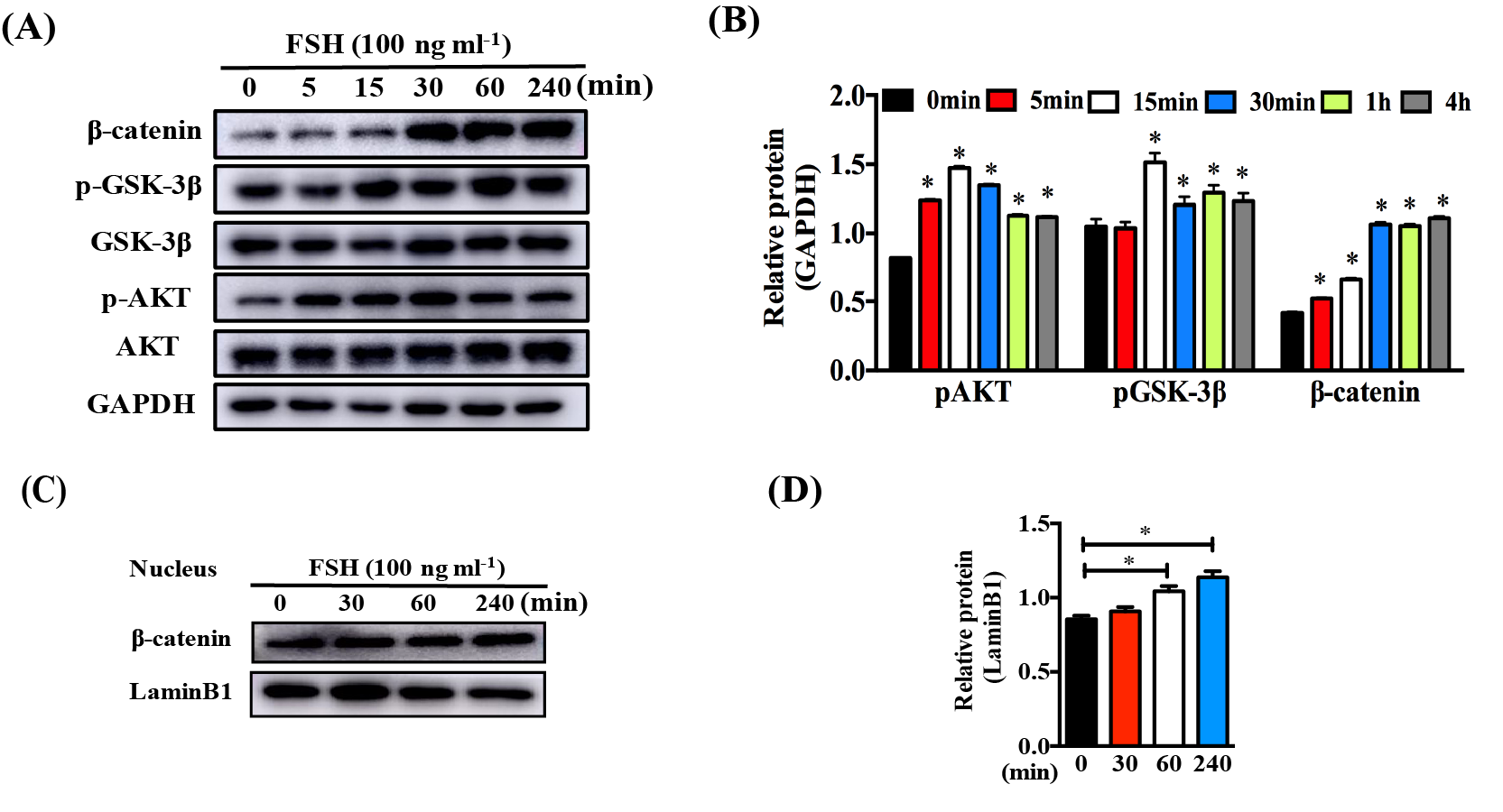

Supplement: Supplementary file 5 [file ACEL-18-e12997-s005.tif]
