# Supplementary material for: Follicle‐stimulating hormone promotes renal tubulointerstitial fibrosis in aging women via the AKT/GSK‐3β/β‐catenin pathway
Source: Aging Cell. 2019 Jun 26;18(5):e12997. doi: 10.1111/acel.12997 (PMC6718534; doi:10.1111/acel.12997)
Supplement: Supplementary file 6 [file ACEL-18-e12997-s006.docx]

Table S1. Characteristics of the participants by quartiles of follicle stimulating hormone

|  | Follicle stimulating hormone, IU/L | | | |  |
| --- | --- | --- | --- | --- | --- |
|  | Q1 | Q2 | Q3 | Q4 |  |
|  | ≤47.30 | 47.31-61.10 | 61.11-78.10 | ≥78.11 | *P for trend* |
| *N* | 772 | 765 | 755 | 763 |  |
| Age, yr | 63(7) | 64(7) | 63(8) | 62(8) | <0.01 |
| GFR, mL/min per 1.73 m^2^ | 84.1(12.8) | 82.2(13.2) | 81.2(13.0) | 78.5(14.8) | <0.001 |
| Creatinine, μmol/L | 66.8(10.7) | 68.5(12.9) | 69.4(10.5) | 73.6(20.0) | <0.001 |
| Total testosterone, nmol/L | 0.69(1.22) | 0.65(0.47) | 0.61(0.43) | 0.56(0.40) | <0.001 |
| Estradiol, pmol/L | 81.2(89.3) | 61.2(63.5) | 54.1(83.8) | 43.7(33.5) | <0.001 |
| Luteinizing hormone, IU/L | 17.4(6.3) | 23.3(7.3) | 27.9(7.9) | 37.7(12.5) | <0.001 |
| Body mass index, kg/m^2^ | 26.3(3.8) | 25.3(3.3) | 24.4(3.3) | 23.8(3.3) | <0.001 |
| Triglycerides, mmol/L | 1.90(1.54) | 1.71(1.10) | 1.66(1.02) | 1.55(0.82) | <0.001 |
| High density lipoprotein, mmol/L | 1.38(0.32) | 1.45(0.31) | 1.51(0.31) | 1.57(0.32) | <0.001 |
| Low density lipoprotein, mmol/L | 3.30(0.86) | 3.35(0.85) | 3.28(0.80) | 3.34(0.79) | 0.78 |
| Total cholesterol, mmol/L | 5.47(1.42) | 5.48(1.12) | 5.46(1.00) | 5.55(0.99) | 0.19 |
| Current smoker, % | 3.9 | 2.6 | 4.6 | 2.0 | 0.19 |
| Diabetes, % | 27.7 | 20.3 | 16.8 | 11.7 | <0.001 |
| Hypertension, % | 64.7 | 61.5 | 58.9 | 52.2 | <0.001 |
| Dyslipidemia, % | 47.5 | 40.8 | 35.8 | 34.1 | <0.001 |

The data are summarized as the mean (standard deviation) for continuous variables, or as number with proportion for categorical variables. *P* for trend was calculated by ANOVA and Chi-square test.
