# Supplementary material for: Follicle‐stimulating hormone promotes renal tubulointerstitial fibrosis in aging women via the AKT/GSK‐3β/β‐catenin pathway
Source: Aging Cell. 2019 Jun 26;18(5):e12997. doi: 10.1111/acel.12997 (PMC6718534; doi:10.1111/acel.12997)
Supplement: Supplementary file 7 [file ACEL-18-e12997-s007.docx]

| Table S2. Primer used in quantitative real-time PCR | | |
| --- | --- | --- |
| Gene Accession Number Sequences | | |
| **Mouse** | | |
| PAI-1 | NM_008871.2 | F: 5'-GGACACCCTCAGCATGTTCA-3'  R: 5'-TCTGATGAGTTCAGCATCCAAGAT-3' |
| Fibronectin | NM_010233.2 | F: 5'-AGACCATACCTGCCGAATGTAG-3'  R: 5'-GAGAGCTTCCTGTCCTGTAGAG-3' |
| Collagen IV | NM_009931.2 | F: 5'-TACCTGCCACTACTTCGCTAAC-3'  R: 5'-CGGATGGTGTGCTCTGGAAG-3' |
| TNF-α | NM_013693.3 | F:5'- CCTGTAGCCCACGTCGTAG-3'  R:5'- GGGAGTAGACAAGGTACAACCC-3' |
| Il-1β | NM_008361.4 | F: 5'-GAAATGCCACCTTTTGACAGTG-3'  R: 5'-TGGATGCTCTCATCAGGACAG-3' |
| iNOS | NM_010927.4 | F: 5'-ACATCGACCCGTCCACAGTAT-3'  R: 5'-CAGAGGGGTAGGCTTGTCTC-3' |
| ROS-1 | NM_011282.2 | F: 5'-AGCTGGACTTTCACGGAGAC-3'  R: 5'-GGTGGGGAATAAAGATGCAGTT-3' |
| Fizz1 | NM_020509.3 | F: 5'-CCAATCCAGCTAACTATCCCTCC-3'  R: 5'-ACCCAGTAGCAGTCATCCCA-3' |
| MR | NM_001083906.2 | F: 5'-CTCTGTTCAGCTATTGGACGC-3'  R: 5'-TGGCACTCCCAA ACATAATTTGA-3' |
| β-actin | NM_007393.5 | F: 5'-GGCTGTATTCCCCTCCATCG-3'  R: 5'-CCAGTTGGTAACAATGCCATGT-3' |
| **Human** | | |
| Collagen IV | NM_001836.3 | F: 5'-CCAGGGGTCGGAGAGAAAG-3'  R: 5'-GGTCCTGTGCCTATAACAATTCC-3' |
| PAI-1 | NM_000602.4 | F: 5'-ACCGCAACGTGGTTTTCTCA-3'  R: 5'-TTGAATCCCATAGCTGCTTGAAT-3' |
| Fibronectin | NM_212482.2 | F: 5'-CGGTGGCTGTCAGTCAAAG-3'  R: 5'-AAACCTCGGCTTCCTCCATAA-3' |
| FSHR | NM_000145.3 | F:5'- TTTTGCAGCTGCCCTCTTT-3'  R:5'- GCACTGTGAGGTAGATGTGGATA-3' |
| β-actin | NM_001101.4 | F: 5'-AAGGTGACAGCAGTCGGTT-3'  R: 5'-TGTGTGGACTTGGGAGAGG-3' |
